# DNA barcoding of reef‐associated fishes of Saint Martin's Island, Northern Bay of Bengal, Bangladesh

**DOI:** 10.1002/ece3.10641

**Published:** 2023-10-22

**Authors:** Kazi Ahsan Habib, Md. Jayedul Islam, Md. Nazmus Sakib, Parsha Shanjana Brishti, Amit Kumer Neogi

**Affiliations:** ^1^ Department of Fisheries Biology and Genetics, Faculty of Fisheries, Aquaculture and Marine Science Sher‐e‐Bangla Agricultural University Dhaka Bangladesh; ^2^ Aquatic Bioresource Research Lab, Department of Fisheries Biology and Genetics Sher‐e‐Bangla Agricultural University Dhaka Bangladesh

**Keywords:** 16S rRNA, COI, mitochondrial DNA, northern Bay of Bengal, reef‐associated fish

## Abstract

This study employs the DNA barcoding approach to make a molecular taxonomic catalog of reef fishes of Saint Martin's Island (SMI), an ecologically critical area (ECA), and Marine Protected Area (MPA) in Bangladesh. DNA barcoding, along with morphological analysis, confirmed 84 reef‐associated fish species in SMI belonging to 16 orders, 39 families, and 67 genera. A total of 184 sequences were obtained in this study where 151 sequences (534–604 bp) of 81 species were identified from the COI barcode gene and 33 sequences (609 bp) of 19 species from the 16S rRNA gene region which were submitted to the GenBank and Barcode of Life Data System (BOLD). Among these sequences, 70 sequences of the COI gene and 16 sequences of 16S rRNA gene region from 41 species were submitted for the first time into the GenBank from Bangladesh. For molecular characterization analysis, another 37 sequences of 15 reef fish species of SMI were added from previous studies, making a total of 221 DNA sequences which comprised 179 sequences of 96 species for the COI gene and 42 sequences of 26 species for the 16S rRNA gene region. The COI sequences contain 145 haplotypes with 337 polymorphic sites, and the mean genetic distances within species, genera, and families were calculated as 0.34%, 12.26%, and 19.03%, respectively. On the contrary, 16S rRNA sequences comprised 31 haplotypes with 241 polymorphic sites, and the mean genetic divergences within species, genera, and families were 0.94%, 4.72%, and 12.43%, respectively. This study is a significant contribution to the marine biodiversity of Bangladesh which would facilitate the assessment of species diversity for strategizing management action. It is also an important input to the DNA barcode library of reef fishes of the northern Bay of Bengal.

## Graphical abstract

Figures 2 and 6 consist of all the species that were sequenced and identified in this study. It also shows the phylogenetic relationship of the studied species.

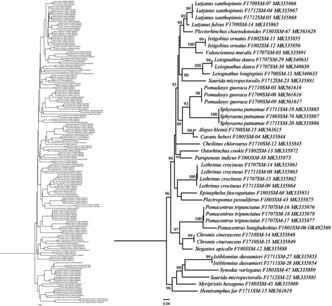

## 1 INTRODUCTION

The Bay of Bengal covers 2,172,000 sq. km in the northeastern Indian Ocean, representing about 12% of the world's coral reefs (BOBLME, 2011). Heavy sediment discharge of the Ganges‐Brahmaputra‐Meghna River system, representing about 6% of the world's total sediment input into the oceans by rivers along with a lack of hard substrate limit the development of viable coral communities and coral reefs in the north and northeast Bay of Bengal (Rajasuriya, 2002; Sheppard, 2000; Spalding et al., 2001). In these relatively turbid coastal waters of the northeastern Bay of Bengal, about 9 km south of the mouth of the Naf River, there is a dumbbell‐shaped small rocky island namely Saint Martin's Island (SMI). Including the rocky platforms extending into the sea, the total area of the island is about 12 sq. km. The island is located on a shallow continental shelf with a maximum depth of 25 m. Its shallow‐water marine habitats comprise rocky and sandy intertidal, intertidal rock pools, offshore lagoons, rocky and sandy subtidal, and offshore soft‐bottom habitats. Shoreline habitats are sandy beaches and dunes, scattered rocks, and coral boulders, which are also found on the interior of the island (Alam & Hassan, 1997; Tomascik, 1997). The rocky habitats that support diverse scleractinian coral communities, and seaweed‐seagrass beds extend up to 200 m offshore from the lower intertidal. There are only a few examples worldwide where coral‐algal communities dominate rocky reefs (Hossain & Islam, 2006).

SMI is the only island in Bangladesh that supports coral communities with diversified reef‐associated flora and fauna. The island was declared an Ecologically Critical Area (ECA) in 1999 under a section of the Bangladesh Environment Conservation Act, 1995 (Department of Environment, 2015) and as a Marine Protected Area (MPA) by the Bangladesh government in 2022. Tomascik (1997) reported 86 species of reef‐associated fishes from SMI island, Thompson and Islam (2010) listed 89 species of reef fish and BOBLME (2015) recorded 55 species of reef‐associated fish. All of these numbers were counted based on photographic records. In the last few years, several reef fish species have been added to the country's marine fish inventory (Akash et al., 2021; Fuad et al., 2021; Habib & Islam, 2020; Habib, Islam, Nahar, Neiogi, & Fraser, 2021; Habib, Islam, Nahar, & Neogi, 2020; Habib, Islam, Nahar, Rashed, et al., 2021; Habib, Islam, Neogi, et al., 2020; Habib, Neogi, Islam, & Nahar, 2019; Islam et al., 2020, 2021; Islam & Habib, 2020; Saha et al., 2018, 2021; Sharifuzzaman, Fuad, et al., 2021; Sharifuzzaman, Rubby, et al., 2021; Siddiqueki et al., 2021). All of these species were identified based on morphological analysis. However, only a few studies used DNA barcoding tools for identification such as Saha et al. (2018, 2021), Habib, Islam, Nahar, and Neogi (2020); Habib, Islam, Neogi, et al. (2020); Habib, Islam, Nahar, Rashed, et al. (2021); Habib, Islam, Nahar, Neiogi, and Fraser (2021), and Islam et al. (2021).

Traditionally, fishes are identified based on morphological features. However, due to high diversity, dramatic phenotypic changes during development, variability in their morphological colouration, sexual dimorphism, or ontogenetic development in many cases, reef fish species are sometimes difficult to identify by using morphological characteristics alone (Duarte et al., 2017; Hubert et al., 2010; Leis & Carson‐Ewart, 2004; Victor et al., 2009). DNA barcoding technique, which involves sequencing approximately 650 base pairs of the mitochondrial gene cytochrome oxidase subunit I (COI), has recently emerged to support species identifications for different taxonomic groups and uncover biological diversity and also proved as a reliable tool for species conservation (Floyd et al., 2002; Hebert et al., 2003; Tautz et al., 2003; Ward et al., 2005). It is an effective tool to detect all life stages including eggs, larvae, juveniles (Hubert et al., 2008, 2010, 2015), sexually dimorphic species or those with large phenotypic plasticity and cryptic species (Sekino & Yamashita, 2013; Winterbottom et al., 2014) that are widely distributed in marine systems, especially in coral reef‐associated organisms (Hubert et al., 2012). This tool is also useful for detecting those species that are often misidentified or difficult to detect using traditional taxonomic methods (Becker et al., 2015; Burghart et al., 2014; Knowlton et al., 1993; Knowlton, 2000; Ko et al., 2013; Lee & Kim, 2014; Lin et al., 2016). This advanced molecular marker is also capable of providing additional information to identify unique and new species from marine ecosystems and reveals undisclosed biodiversity than previously estimated (Brasier, 2017; Habib, Neogi, Islam, & Nahar, 2019; Jaafar et al., 2012). Thus, the DNA barcoding method now represents the largest effort to catalog biodiversity using molecular approaches, especially for a diverse group of individuals.

Further, the mitochondrial 16S ribosomal RNA (16S rRNA) gene is highly conserved in some animal taxa. This 16S rRNA gene region has been used for the identification of different organisms including marine invertebrates and fishes (Chakraborty & Iwatsuki, 2006; Habib et al., 2017; Hernández et al., 2019; Hossain et al., 2019; Li et al., 2008; Lv et al., 2014; Vences et al., 2005; Zhang & Hanner, 2012; Zheng et al., 2014). Although the absolute rate of change in the 16S rRNA gene sequence is not known, it does mark the evolutionary distance and relatedness of organisms (Kimura, 1980; Pace, 1997; Rajendhran & Gunasekaran, 2011; Thorne et al., 1998). Thus, the 16S rRNA can assist in species identification along with COI.

In recent years, DNA barcoding has been frequently used to assess the coral‐associated fish diversity in different locations of the Indo‐Pacific region such as Weh Island (Fadli et al., 2020) and Ambon Harbor (Limmon et al., 2020) of Indonesia, Mischief Reef of Nansha Islands (Shan et al., 2021). In Bangladesh, some DNA barcoding studies of fishes of both marine and freshwater habitats have been accomplished in the last few years such as Ahmed et al. (2019), Rahman et al. (2019), Ahmed et al. (2021), and Habib, Neogi, Rahman, Oh, et al. (2021). However, there is a lack of specific studies focusing exclusively on the DNA barcoding of reef‐associated fish species in Bangladesh. Considering the importance of ECA and MPA of Bangladesh, as well as the northern Bay of Bengal, the present study aims to assess the diversity and make an updated inventory of reef‐associated fishes of SMI through DNA barcoding, and to build a reference library of DNA barcode data for reef‐associated fishes of Bangladesh. This kind of molecular study particularly focusing on reef fishes has rarely been conducted not only in Bangladesh but also in the entire Bay of Bengal region.

## 2 METHODOLOGY

### 2.1 Collection of samples

**FIGURE 1:**
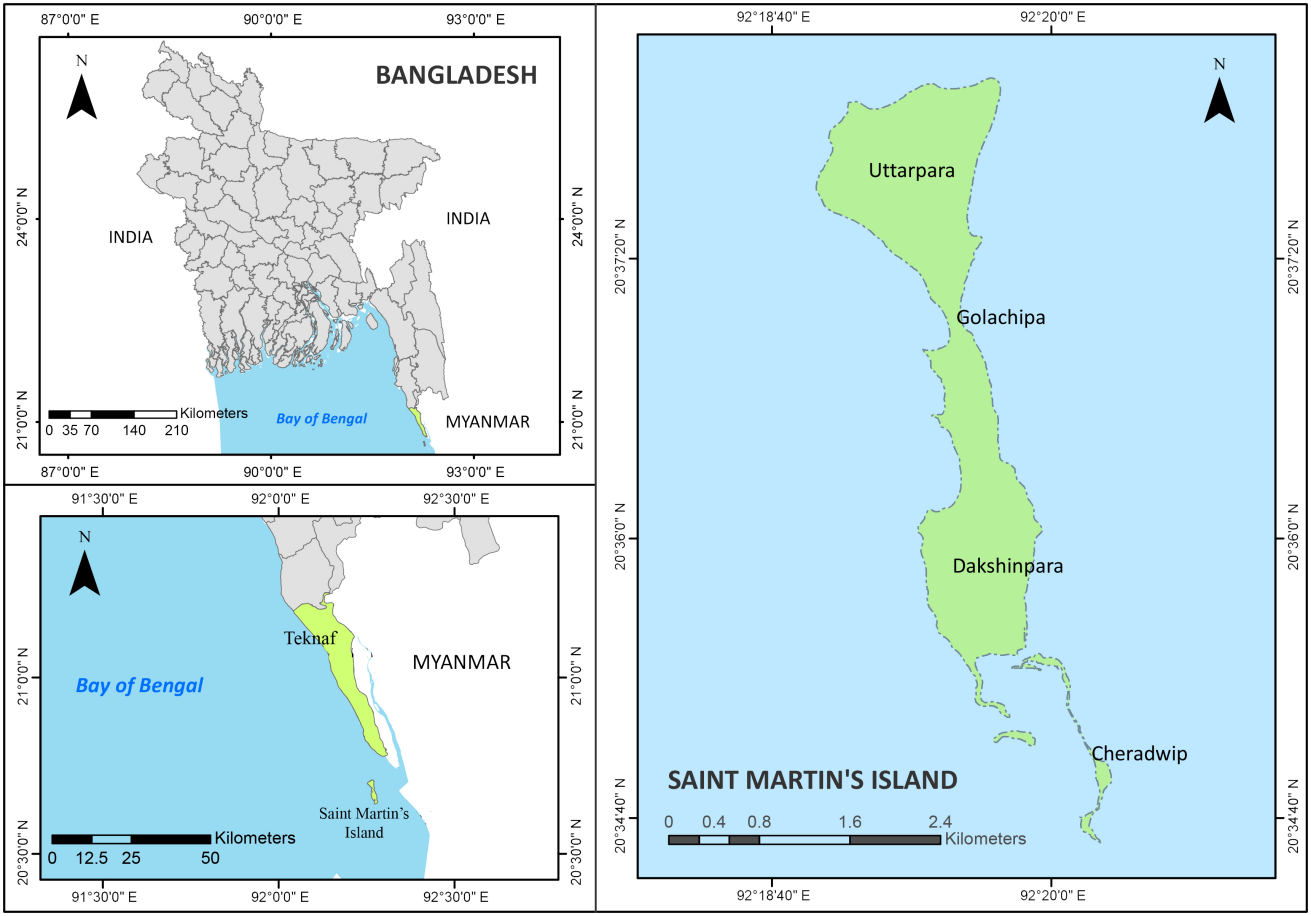
Location of Saint Martin's Island (Subdistrict: Teknaf, District: Cox's Bazar) in Bangladesh.

Specimens of fish were collected at landing from local fishermen or traders of SMI between May 2017 and July 2019 (Figure 1). As per the provided information by local fishermen, they were fished using hook and line and gill net set on or around the submerged rock surrounding the island. After tagging, the collected samples were photographed in the field for the best living colour representation. Then it was transferred and stored in the Aquatic Bioresource Research Lab. (ABR Lab.), Department of Fisheries Biology and Genetics, Sher‐e‐Bangla Agricultural University (SAU), Dhaka, Bangladesh for morphological and molecular analysis. The morphological diagnosis (meristic counts and proportional measurements) of collected specimens was performed according to Carpenter and Niem (1999a, 1999b, 2001a, 2001b), Allen et al. (2003), Rahman et al. (2009); Allen and Erdmann (2012), Psomadakis et al. (2019); Froese and Pauly (2023). We followed Frick et al. (2023) for the recent valid name of the genus, species, family, and orders. After species identification by morphological study, a small piece of muscle tissue from the fish specimens was cut and stored in a sterile 1.5 mL tube containing 98% alcohol for subsequent molecular work.

### 2.2 Genomic DNA extraction, PCR amplification, and sequencing

Genomic DNA was extracted from the collected muscle tissue using a TIANamp Marine Animals DNA Kit (TIANGEN) following the protocol provided inside the kit box. The concentration of genomic DNA was then measured by a Qubit 3.0 fluorometer. Polymerase chain reaction (PCR) was performed in a 50‐reaction mixture in small reaction tubes (0.2 mL) in a Thermal cycler (2720 Thermal Cycler, Applied Biosystems). The mitochondrial DNA (mtDNA) COI gene fragment of mtDNA was amplified using either the primer set of FishF1 (5′‐TCAACCAACCACAAAGACATTGGCAC‐3*′*) and FishR1 (5′‐TAGACTCTGGGTGG CCAAAGAATCA‐3*′*) (Ward et al., 2005) or FishF2 (5′‐TCGACTAATCATAAAGATAT CGGCAC‐3′) and FishR2 (5′‐CTTCAGGGTGACCGAAGAATCAGAA‐3′) (Ward et al., 2005). The 16S rRNA sequences were amplified using the primer set 16Sar (5′‐CGCCTGTTTATCAAAAACAT‐3*′*) and 16Sbr‐3*′* (5′‐CCGGTCTGAACTCAGATCACGT‐3*′*) (Palumbi, 1996). The PCR profile consisted of a preheating at 95°C for 2 min followed by 35 cycles of denaturation at 95°C for 1 min, annealing at 54°C for the COI region or at 52°C for the 16S rRNA gene for 40 s, extension at 72°C for 1 min, and completion with a final extension at 72°C for 10 min. After successful PCR, every sample was visualized on 1% agarose gel (EZ‐Vision® In‐Gel Solution, USA) stained with ethidium bromide in the gel documentation chamber (Model: Syngene InGenius^3^). The flow UV‐ray is kept on to watch the band in the connected computer by using GeneSys software. PCR samples with a single and clear visible band were purified with the PCR Purification Kit (TIANGEN‐ Universal DNA Purification Kit) for sequencing. The concentration of the purified DNA was estimated with the help of a Qubit 3.0 fluorometer. Sequencing was conducted with the same PCR primers by the Sanger method with an automated sequencer (ABI 3730 × 1 DNA analyzer) at Macrogen Inc. (Korea).

### 2.3 Data analysis

The obtained consensus sequences were edited based on the chromatogram peak clarities with the help of Chromas Lit and Geneious 9.0.5 program combined with manual proofreading. Stop codons were checked for COI sequences by Expasy translate tools (Duvaud et al., 2021). The sequences were aligned using ClustalW in MEGA 7.0 software and then matched using the BLAST search engine provided by NCBI and the Bold database. The consensus sequences obtained from all specimens through DNA sequencing of both COI and 16S rRNA gene regions were submitted to the BOLD system (project code: SAU) and NCBI GenBank (accession numbers given in Table 1) which are accessible to all researchers. In the data analysis, we also added 37 sequences (28 sequences of COI and 9 sequences of 16 s rRNA gene region) of 15 coral‐associated fishes of SMI previously reported in the GenBank from different studies conducted in ABR Lab. (Reference given in the “source of sequences” column of Table 1).

**TABLE 1:** GenBank accession number of mitochondrial COI and 16S rRNA sequences used in the present study.

Sl No.	Order	Family	Species	GenBank accession no. (mtCOI)	GenBank accession no. (16S rRNA)	Source of sequences
1	Myliobatiformes	Dasyatidae	*Neotrygon indica*	MK340668		This study
2	Siluriformes	Plotosidae	*Plotosus lineatus* ^b^	MN458369, MN458370		This study
3	Aulopiformes	Synodontidae	*Saurida micropectoralis*	MK340700, MK340701	MK335881, MK561622	This study
4		Synodontidae	*Synodus variegatus*	MK340725	MK335889	This study
5		Synodontidae	*Trachinocephalus myops*	MK340735, MK340736		This study
6	Holocentriformes	Holocentridae	*Myripristis hexagona* ^b^	MK340664	MK335869	This study
7		Holocentridae	*Sargocentron rubrum* ^b^	MK340697, MK340698, MK340699		This study
8	Syngnathiformes	Dactylopteridae	*Dactyloptena orientalis* ^b^	MK340606, MK340607		This study
9		Mullidae	*Mulloidichthys vanicolensis* ^b^	MT374171		This study
10		Mullidae	*Parupeneus indicus* ^b^	MK340674	MK335873	This study
11		Mullidae	*Upeneus tragula* ^b^	MK340744		This study
12	Kurtiformes	Apogonidae	*Lepidamia kalosoma*	MK340634, MK560520, MT379891		Habib, Neogi, Rahman, Oh, et al. (2021)
13		Apogonidae	*Ostorhinchus cookii*	MK340670	MK335872	Habib, Islam, Nahar, Rashed, et al. (2021); Habib, Neogi, Rahman, Oh, et al. (2021); Habib, Islam, Nahar, Neiogi, and Fraser (2021)
14	Gobiiformes	Gobiidae	*Amblyeleotris downingi*	MK340584		Islam et al. (2021)
15		Gobiidae	*Cryptocentrus cyanotaenia*	MK561627		This study
16		Gobiidae	*Istigobius ornatus*		MK335855, MK335856	This study
17		Gobiidae	*Valenciennea muralis*	MK340745, MK340746	MK335891	Islam et al. (2021)
18	Carangiformes	Menidae	*Mene maculata*	MK340663		This study
19		Carangidae	*Alectis indica* ^b^	MK340580, MK340581, MT375170		This study
20		Carangidae	*Alepes kleinii*	MK340582, MK340583	MK561615	This study
21		Carangidae	*Caranx heberi*	MK340591	MK335844	This study
22		Carangidae	*Caranx sexfasciatus*	MK340592, MK340593, MK340594, MK340595		This study
23		Carangidae	*Elagatis bipinnulata*	MZ706946		This study
24		Carangidae	*Gnathanodon speciosus*	MK340622		This study
25		Carangidae	*Megalaspis cordyla*	MK340662		This study
26		Carangidae	*Scomberoides commersonnianus*	MK340708, MK340709		This study
27		Carangidae	*Scomberoides lysan* ^b^	MK340710		This study
28		Carangidae	*Scomberoides tol*	MK340711		This study
29		Carangidae	*Seriolina nigrofasciata* ^b^	MK340712		This study
30		Carangidae	*Ulua mentalis* ^b^	MK340738		This study
31		Lactariidae	*Lactarius lactarius* ^b^	MK340629		This study
32		Sphyraenidae	*Sphyraena putnamae* ^b^	MK340720, MK340721, MK340722, MK340723	MK335885, MK335886, MK335887	This study
33		Echeneidae	*Echeneis naucrates* ^b^	MK340612, MT379900		This study
34	Cichliformes	Pomacentridae	*Abudefduf septemfasciatus* ^b^	MK340573, MK340574, MK340575		This study
35		Pomacentridae	*Abudefduf sordidus* ^b^	MK340576		This study
36		Pomacentridae	*Chromis cinerascens* ^b^	MK340603	MK335848, MK335849	This study
37		Pomacentridae	*Neopomacentrus cyanomos* ^b^	MT374163		This study
38		Pomacentridae	*Pomacentrus bangladeshius*	MK340681, MK340682, MK340683	OK482569	Habib, Islam, Nahar, and Neogi (2020)
39		Pomacentridae	*Pomacentrus tripunctatus* ^b^	MK340684, MK340685, MK340686	MK335876, MK335877, MK335878	This study
40		Pomacentridae	*Plectroglyphidodon apicalis* ^b^	MK340724	MK335888	This study
41		Opistognathidae	*Opistognathus rosenbergii* ^c^	MW940139		This study
42		Opistognathidae	*Opistognathus variabilis* ^b^	MK340669		This study
43	Beloniformes	Belonidae	*Ablennes hians*	MK340570, MK340571		This study
44		Hemiramphidae	*Hemiramphus far*		MK561619	This study
45	Blenniiformes	Blenniidae	*Istiblennius dussumieri*	MK340623, MK340624	MK335853, MK335854	This study
46	Perciformes^a^	Priacanthidae	*Priacanthus tayenus* ^b^	MT379892		This study
47		Lutjanidae	*Caesio cuning* ^b^	MK340588, MK340589, MK340590		This study
48		Lutjanidae	*Lutjanus erythropterus*	MK340640, MK340641		Sarkar et al. (2021)
49		Lutjanidae	*Lutjanus fulvus*	MK340642	MK335865	Sarkar et al. (2021)
50		Lutjanidae	*Lutjanus indicus*	MK340661, MT379889		This study
51		Lutjanidae	*Lutjanus johnii*	MK340643, MK340644		This study
52		Lutjanidae	*Lutjanus fulviflamma*	MT379888		This study
53		Lutjanidae	*Lutjanus lemniscatus*	MK340645, MK340646, MK340647, MK340648		This study
54		Lutjanidae	*Lutjanus lunulatus* ^b^	MK340649, MK340650, MK340651		This study
55		Lutjanidae	*Lutjanus lutjanus*	MK340652, MK340653, MK340654		This study
56		Lutjanidae	*Lutjanus rivulatus*	MK340658, MK340659, MK340660		This study
57		Lutjanidae	*Lutjanus xanthopinnis*	MK340655, MK340656, MK340657	MK335866, MK335867, MK335868	This study
58		Lutjanidae	*Pinjalo pinjalo* ^b^	MT379894		This study
59		Haemulidae	*Plectorhinchus macrospilus*	MK340677	MK561628	Habib, Islam, Nahar, Rashed, et al. (2021)
60		Haemulidae	*Plectorhinchus pictum*	MK340608, MK340609, MT379897		Habib, Islam, Nahar, Rashed, et al. (2021)
61		Haemulidae	*Pomadasys andamanensis*	MK340687		Habib, Islam, Nahar, Rashed, et al. (2021)
62		Haemulidae	*Pomadasys guoraca*	MT375172, MK340689, MK340690, MK340691	MK561616, MK561617, MK561618	Habib, Islam, Nahar, Rashed, et al. (2021)
63		Haemulidae	*Pomadasys maculatus*	MK340692, MK340693, MT374170		Habib, Islam, Nahar, Rashed, et al. (2021)
64		Sparidae	*Acanthopagrus berda*	MK340577, MK340578		This study
65		Lethrinidae	*Lethrinus crocineus*	MK340635, MK340636, MK340637, MK340638, MK340639, MT379898, MT379899	MK335861, MK335862, MK335863, MK335864	This study
66		Nemipteridae	*Scolopsis vosmeri*	MK340705, MK340706, MK340707		This study
67	Perciformes	Serranidae	*Cephalopholis boenak*	MK340596		This study
68		Serranidae	*Cephalopholis formosa*	MK340597, MK340598, MK340599		This study
69		Serranidae	*Epinephelus coioides* ^b^	MT379893		This study
70		Serranidae	*Epinephelus erythrurus* ^b^	MK340613, MK340614, MK340615, MK340616		This study
71		Serranidae	*Epinephelus fuscoguttatus* ^b^	MK340617	MK335851	This study
72		Serranidae	*Epinephelus quoyanus* ^b^	MT379895, MT379896		This study
73		Serranidae	*Plectropomus pessuliferus*	MK340678	MK335875	Islam et al. (2020)
74		Labridae	*Bodianus neilli*	MK340586, MK340587		This study
75		Labridae	*Cheilinus chlorourus* ^b^		MK335845	This study
76		Labridae	*Thalassoma lunare* ^b^	MK340732, MK340733, MK340734		This study
77		Scaridae	*Chlorurus rhakoura*	MK560524		Habib, Islam, Neogi, et al. (2020)
78		Scaridae	*Scarus ghobban*	MK340703		Habib, Islam, Neogi, et al. (2020)
79		Scorpaenidae	*Scorpaenodes guamensis* ^b^	MW940140		This study
80		Platycephalidae	*Platycephalus indicus* ^b^	MT374176, MT374177		This study
81	Centrarchiformes	Kyphosidae	*Kyphosus cinerascens* ^b^	MK340627, MK340628		This study
82		Terapontidae	*Terapon theraps*	MK340729, MK340730		This study
83	Acropomatiformes	Pempheridae	*Pempheris malabarica*	MT375173		This study
84	Acanthuriformes	Pomacanthidae	*Pomacanthus annularis*	MK340680		This study
85		Drepaneidae	*Drepane longimana*	MK340610, MK340611		This study
86		Chaetodontidae	*Chaetodon decussatus*	MK340600		This study
87		Ephippidae	*Platax teira*	MK340675, MK340676		This study
88		Leiognathidae	*Karalla daura* ^b^	MK340630, MK340631, MK340632	MK335857, MK335858	This study
89		Leiognathidae	*Leiognathus longispinis*	MT374174, MK560522, MK340633	MK335860	This study
90		Siganidae	*Siganus canaliculatus*	MK340713, MK340714, MK340715		This study
91		Siganidae	*Siganus javus*	MK340716		This study
92		Siganidae	*Siganus vermiculatus*	MK340717		This study
93		Acanthuridae	*Acanthurus mata*	MK560531		This study
94		Acanthuridae	*Acanthurus xanthopterus*	MK340579		This study
95	Tetraodontiformes	Tetraodontidae	*Chelonodontops patoca* ^b^	MK560528		This study
96		Ostraciidae	*Tetrosomus gibbosus* ^b^	MK340731		This study
97		Balistidae	*Balistoides viridescens* ^b^	MK560530		This study
98		Balistidae	*Canthidermis maculata* ^b^	MW940138, MZ706943, MZ706944, MZ706945		This study
99		Balistidae	*Sufflamen fraenatum* ^b^	MK560529		This study

^a^ Denotes “sedis mutabilis” (Fricke et al., 2023), that is, uncertain Order level status of the Families mentioned within (as per recent phylogenetic studies), but herein tentatively placed under Perciformes.

^b^ First time submitted to GenBank from Bangladesh.

^c^ First‐ever contribution to GenBank.

Pairwise genetic distances at different taxonomic levels (within species, within genera, and within families) and Barcoding Gap Analysis were calculated by the Kimura‐2‐parameter (K2P) model and Kalign multiple species alignment (Lassmann & Sonnhammer, 2005) using Sequence Analysis Engine of BOLD (http://www.boldsystems.org/). Phylogenetic analysis was performed using maximum likelihood (ML) methods through IQ‐TREE v1.6.12 (Nguyen et al., 2015; Trifinopoulos et al., 2016). The robustness of the phylogenetic relationships was evaluated by bootstrap analysis with 100,000 replications (Felsenstein, 1985). We used the evolutionary model GTR + F + I + G4 as the best‐fit model, which was selected by Model Finder (Kalyaanamoorthy et al., 2017) applying the Bayesian information criterion. The Kimura‐2 parameter (K2P) distance model (Kimura, 1980) was used for calculating the genetic distance among the sequences using MEGA‐7. The ML tree was visualized using FigTree v1.4.3 (Rambaut & Drummond, 2016) and edited by Adobe Illustrator. Sequence composition and GC% in different codon positions of COI barcode region and overall GC% of 16S rRNA sequences were measured by the BOLD system analyzer version 3. The nucleotide diversity, number of polymorphic sites, and haplotype diversity were obtained using the program ARLEQUIN (version 3.5; Schneider et al., 2000).

## 3 RESULTS

Morphological and molecular analyses confirmed a total of 84 reef‐associated fish species belonging to 16 orders, 39 families, and 67 genera in the present study. Among the identified species, six species, for example, *Canthidermis maculata* (Bloch, 1786), *Epinephelus fuscoguttatus* (Forsskål 1775), *Plectroglyphidodon apicalis* (De Vis, 1885), *Synodus variegatus* (Lacepède, 1803), *Opistognathus variabilis* Smith‐Vaniz, 2009, and *Opistognathus rosenbergii* Bleeker, 1856 are new distributional records in Bangladesh. A total of 184 sequences (COI and 16S rRNA) were obtained in the study where 151 sequences of 81 species were attained from the COI gene and 33 sequences of 19 species from the 16S rRNA gene region. Among 81 fish species, 16 species were common from where both COI and 16S rRNA gene regions were sequenced. Among the submitted sequences, 86 sequences (70 sequences from the COI gene and 16 sequences from the 16S rRNA gene region) of 41 species were submitted for the first time into the GenBank from Bangladesh (Table 1).

A total of 179 COI barcode sequences of reef fishes of SMI were used for molecular characterization and phylogenetic analyses where 151 sequences of 81 species were obtained from the present study and 28 sequences of 15 species were collected from previous studies (Table 1). After editing and aligning all of these COI sequences the length of the consensus sequences was 534–604 bp.

**FIGURE 2:**
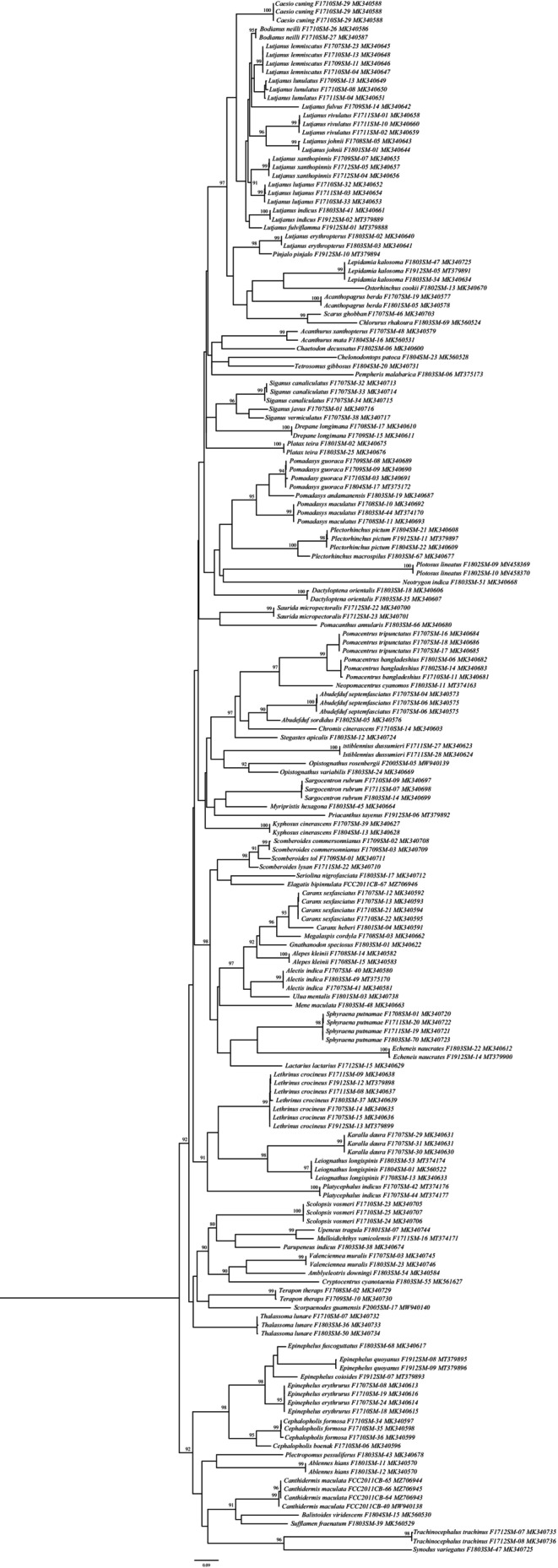
Maximum‐likelihood tree constructed for COI gene sequences of 179 sequences of 96 species of SMI used in the present study. Values of bootstrap support of >70% are shown above branches. The scale bar indicates several nucleotide substitutions per site.

**FIGURE 3:**
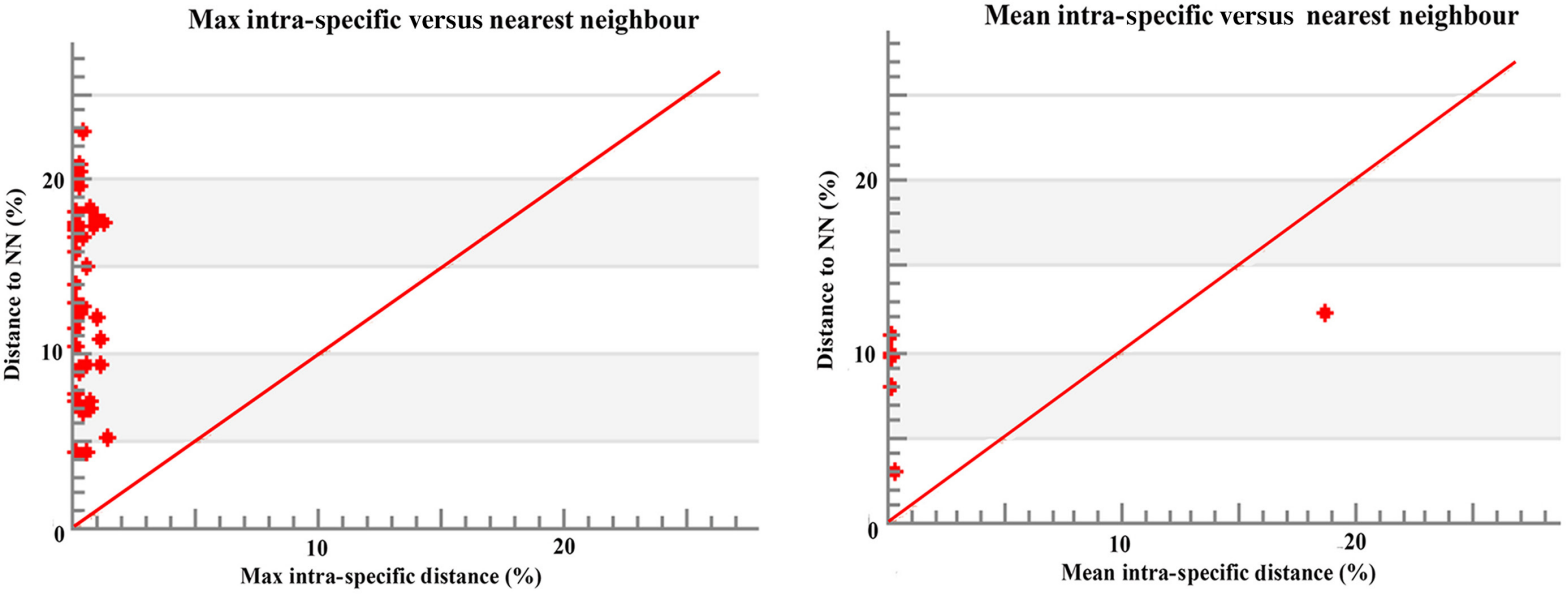
Maximum and mean intraspecific divergence (% K2P) in the barcode region of COI plotted against nearest neighbor distance (% K2P) for examined species in this study. All comparisons had a barcode gap based on the positions of all points above the red line.

In the phylogenetic tree, COI barcode sequences discriminated all the species and clustered the similar species with significant bootstrap values of 80%–100% under the same nodes (Figure 2). The assessment of species identities with previously known sequences and closely related species in GenBank databases generated 98%–100% identities indicating the effectiveness of COI sequences in providing species‐level resolution. In addition, Barcoding Gap Analysis showed that no species lacked a barcode gap (intraspecific K2P distance ≥ interspecific), no species with high intraspecific distance (>2%), and no species with low distance to other species (≤2%) which indicates that all of the studied species identified by the DNA barcode approach. The mean distance to the nearest neighbor (NN) was 14.18 ± 0.05% (mean ± SD; Figure 3).

**FIGURE 4:**
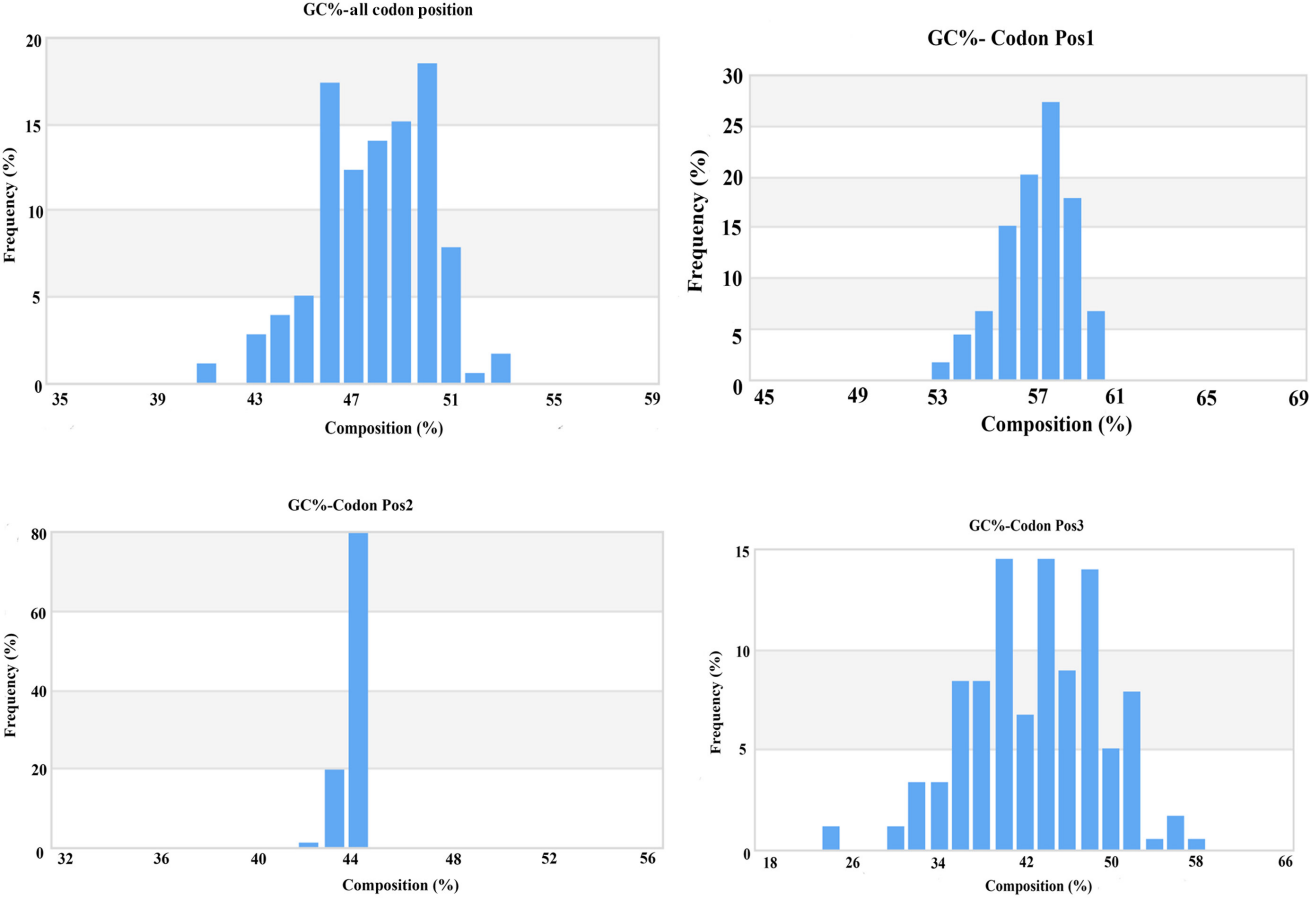
Codon composition of 179 COI barcodes for reef‐associated fish of SMI.

The 179 COI sequences of 96 species comprised 145 haplotypes with 337 polymorphic sites. A total of 82 indels were found. The nucleotide diversity was calculated as 0.19 ± 0.01 (mean ± SD) and the haplotype diversity was 0.99 ± 0.00 (mean ± SD) for the COI sequences. Parsimony informative sites of two, three, and four variants were 103, 25, and 104. The number of transitions and transversion of studied COI sequences were 392 and 167, respectively. The estimated Transition/Transversion bias (R) was 1.99. Substitution patterns and rates were estimated using the Kimura 2‐parameter model. Rates of different transitional substitutions are exposed in bold and the transversional substitutions are exposed in italic in Table 2. The overall mean nucleotide base frequencies observed for 179 COI sequences were 23.73 ± 0.09% (mean ± SD), 28.91 ± 0.12% (mean ± SD), 28.88 ± 0.14% (mean ± SD) and 18.48 ± 0.07% (mean ± SD) for adenine (A), thymine (T), cytosine (C) and guanine (G), respectively. The base composition analysis for the COI sequence showed that the average T content was the highest and the average G content was the lowest; the mean GC content was 47.36%. The GC contents at the first, second, and third codon positions for the 179 sequences of 96 reef‐associated fishes were 56.94%, 43.03%, and 42.08%, respectively. The distribution of GC composition by all of the 3 codon positions is given in Figure 4.

**TABLE 2:** Estimation of substitution matrix of COI sequences of maximum likelihood.

From\to	A	T	C	G
A	–	*5.27*	*4.96*	**10.27**
T	*4.05*	–	**20.27**	*3.22*
C	*4.05*	**21.52**	–	*3.22*
G	**12.93**	*5.27*	*4.96*	–

**FIGURE 5:**
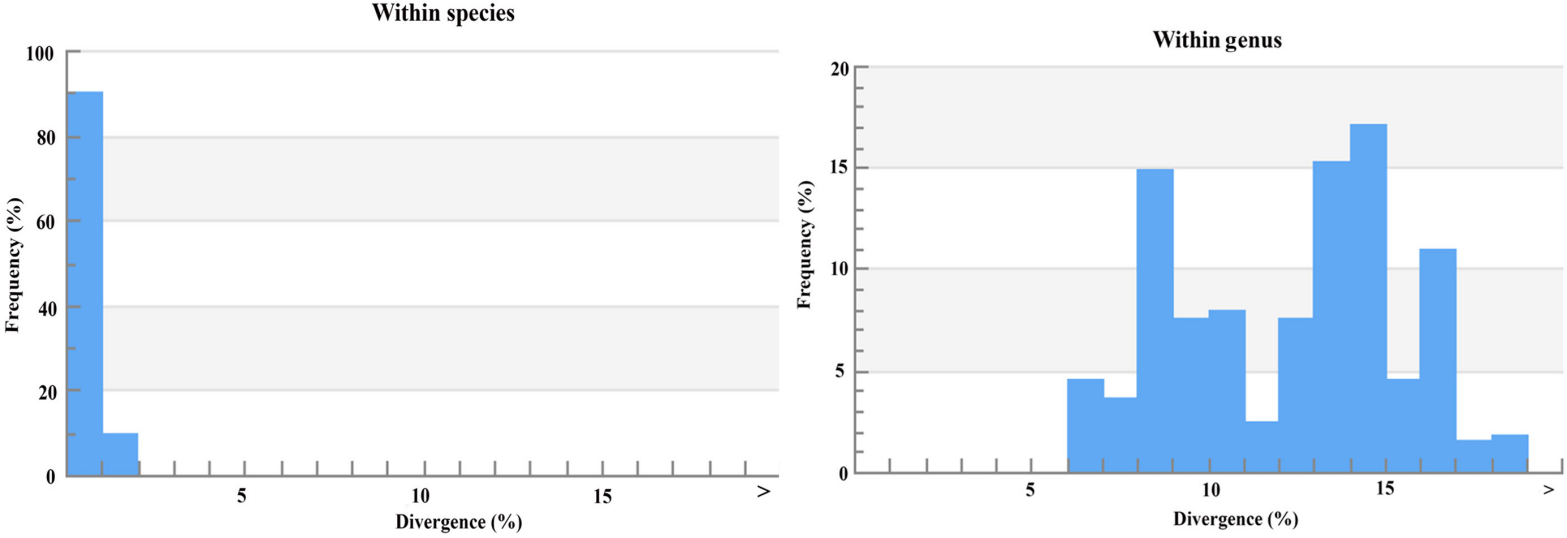
Sequence divergence graph for all COI sequences compared at the species and genus levels.

The overall mean distance of the COI sequences was 23.50 ± 0.01% (mean ± SD). A summary of genetic distances of different taxonomic levels viz., within species, genera, and families based on the Kimura two‐parameter (K2P) distance model is given in Table 3. Minimum genetic distances within species are 0.00% and the maximum is 1.49%; the minimum genetic distance within the genus is 6.05% and the maximum is 18.77%. The minimum genetic distance within the family is 7.37% and the maximum is 25.46%. Sequence divergence of 179 COI barcode sequences compared at the species and genus levels are shown in Figure 5.

**TABLE 3:** The distribution of sequence divergence at each taxonomic level of COI sequences.

	Comparisons	Min Dist. (%)	Mean Dist. (%)	Max Dist. (%)	SE Dist. (%)
Within Species	135	0.00	0.34	1.49	0.00
Within Genus	328	6.05	12.26	18.77	0.01
Within Family	358	7.37	19.03	25.46	0.01

**FIGURE 6:**
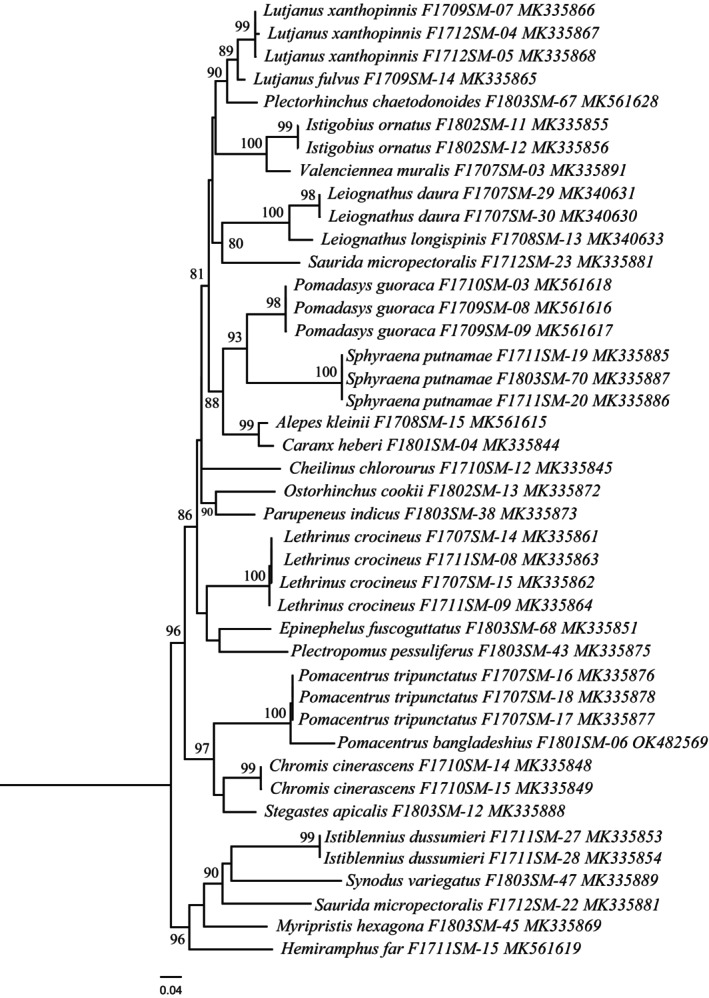
Maximum‐likelihood tree constructed for 16 s rRNA gene sequences of 42 sequences of 26 species of SMI. Values of bootstrap support of >70% are shown above branches. The scale bar indicates the number of nucleotide substitutions per site.

Sequence alignment of 16S rRNA gene regions of the present study after trimming of primer ends yielded 609 bp long nucleotide sequences. A total of 42 sequences of 26 species were used in the molecular characterization and phylogenetic analysis where 33 sequences of 19 species were obtained from the present study and 9 sequences of 7 species were collected from previous studies. In phylogenetic analysis, the sequences discriminated all species clustering the same species under the same nodes with significant bootstrap values of 80%–100% (Figure 6).

The 16S rRNA sequences obtained from 26 species comprised 31 haplotypes with 241 polymorphic sites. The nucleotide diversity was calculated as 0.132 and the haplotype diversity was 0.984 ± 0.009 (mean ± SD). Parsimony informative sites of two, three, and four variants were 84, 54, and 41, respectively. The number of transition and transversion of studied 16S rRNA sequences were 303 and 164, respectively.

The mean genetic distance (%) among all sequences of 16S rRNA was estimated as 15.30 ± 0.01(mean ± SD). The mean nucleotide base compositions were calculated as A = 28.63 ± 0.17% (mean ± SD), T = 22.81 ± 0.19% (mean ± SD), C = 25.47 ± 0.19% (mean ± SD), and G = 23.10 ± 0.12% (mean ± SD). The base composition analysis for the 16S rRNA sequences showed that the average C content was the highest and the average T content was the lowest. The mean GC content was 48.57%.

**FIGURE 7:**
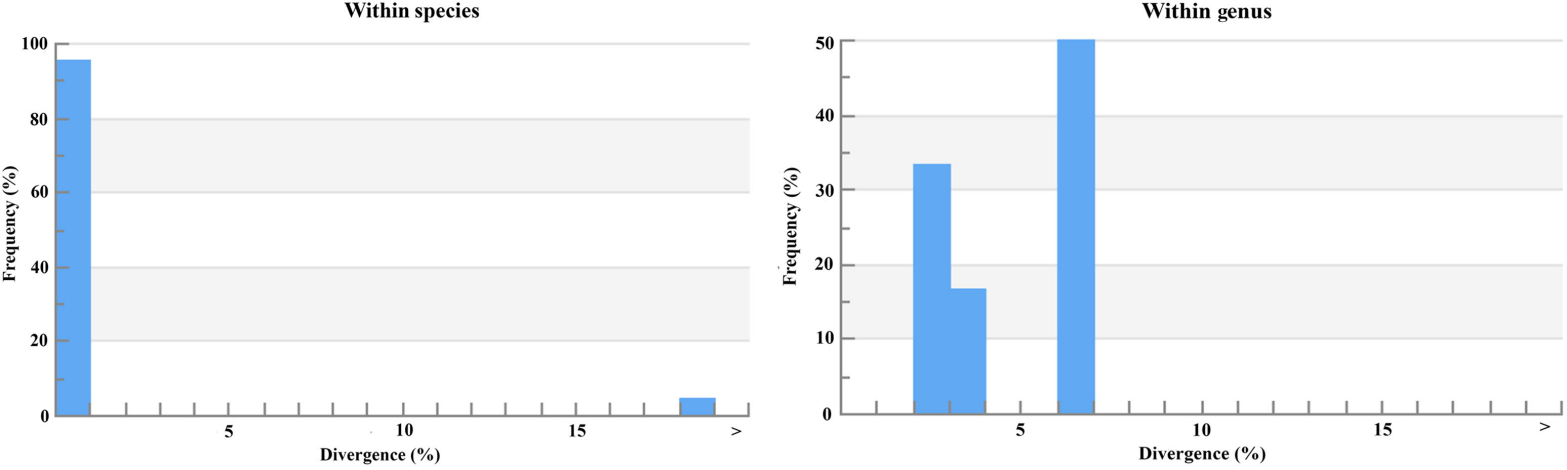
Sequence divergence graph for all 16S rRNA sequences compared at the species and genus levels.

**FIGURE 8:**
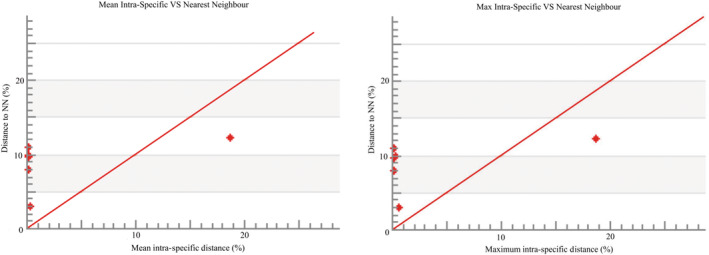
Maximum and mean intraspecific divergence (% K2P) in the barcode region of 16S rRNA plotted against nearest neighbor distance (% K2P) for the examined species in this study. All comparisons had a barcode gap based on the positions of all points above the red line.

A summary of genetic distances of different taxonomic levels viz., within species, within genera, and within families based on the Kimura two‐parameter (K2P) distance model is given in Table 4. Minimum genetic distances within species are 0.00% and the maximum is 6.63%, minimum genetic distance within the genus is 2.95% and the maximum is 18.66%. The minimum genetic distance within the family is 4.29% and the maximum is 22.59%. Sequence divergence of 42 16S rRNA sequences compared at the species and genus levels is shown in Figure 7. Barcoding Gap Analysis showed that 1 species lacks barcode gap (intraspecific ≥ interspecific), 1 species with high intraspecific distance (>2%), and no species with a low distance to another species (≤2%), which indicates that most of the species of the studied species identified by the DNA barcode approach. The mean distance to the nearest neighbor (NN) was 9.23 ± 0.14% (mean ± SD; Figure 8).

**TABLE 4:** The distribution of sequence divergence at each taxonomic level of 16S rRNA sequences.

	Comparisons	Min Dist. (%)	Mean Dist. (%)	Max Dist. (%)	SE Dist. (%)
Within Species	26	0.00	0.94	6.36	0.16
Within Genus	8	2.95	4.72	18.66	0.27
Within Family	24	4.29	12.43	22.59	0.16

The estimated transition/transversion average ratio (R) is 1.51. Substitution patterns and rates were estimated using the Kimura two‐parameter (K2P) model, and rates of different transitional substitutions are given in bold fonts and those of transversional substitutions are given in italics fonts in Table 5.

**TABLE 5:** Estimation of substitution matrix of 16S rRNA sequences of maximum likelihood.

From\to	A	T	C	G
A	–	*4.41*	*4.92*	**11.76**
T	*5.43*	–	**18.88**	*4.74*
C	*5.43*	**16.89**	–	*4.74*
G	**13.47**	*4.41*	*4.92*	–

## 4 DISCUSSION

The present study represents the first molecular survey of the reef‐associated fish fauna of Bangladesh. This study has demonstrated the uses of DNA barcoding to complement the morphological identification of 84 reef fish species from SMI. These DNA barcodes of reef fishes will significantly contribute to making the DNA barcode reference library of marine fishes of Bangladesh and broadly to the global DNA barcode entries. This baseline database is significant for future fisheries management and biodiversity conservation strategy of this MPA.

Through its rapid development over the one‐and‐a‐half‐decade, DNA barcoding has represented a well‐established molecular tool in taxonomic research (Gong et al., 2018). Differences in evolutionary rates provide various DNA barcoding options but make it difficult to find a universal DNA barcode for all species (Gong et al., 2018). Currently, the mitochondrial genes coding COI and 16S rRNA are considered reliable DNA barcodes for the identification of marine species (Habib, Neogi, Rahman, Oh, et al., 2021). DNA barcoding application by mtDNA COI and 16S rRNA gene sequencing together with morphological analysis in some recent studies also revealed several previously unrecognized reef‐associated fish species as new records in SMI of Bangladeshi marine water. For example, Habib, Islam, Nahar, and Neogi (2020) discovered a new species namely *Pomacentrus bangladeshius* Habib, Islam, Nahar, & Neogi, 2020 from SMI. Further, fourteen reef fish species viz. *Amblyeleotris downingi* Randall, 1994, *Apogonichthyoides sialis* (Jordan & Thompson, 1914), *Chlorurus rhakoura* Randall & Anderson, 1997, *Lepidamia kalosoma* (Bleeker, 1852), *Lutjanus erythropterus* Bloch, 1790, *Lutjanus fulvus* Forster, 1801, *Lutjanus indicus* Allen, White & Erdmann, 2013, *Lutjanus xanthopinnis* Iwatsuki, Tanaka & Allen, 2015, *Scarus ghobban* Forsskal, 1775, *Ostorhinchus cookii* (Macleay, 1881), *Plectorhinchus macrospilus* (Satapoomin and Randall, 2000), *Plectropomus pessuliferus* (Fowler, 1904), *Pomadasys guoraca* (Cuvier, 1829), and *Valenciennea muralis* (Valenciennes, 1837) were detected as the first records from the northern Bay of Bengal by Saha et al. (2018), Habib, Islam, Neogi, et al. (2020), Habib, Islam, Nahar, Rashed, et al. (2021), Habib, Islam, Nahar, Neiogi, and Fraser (2021), Islam et al. (2020), Islam et al. (2021), and Sarkar et al. (2021).

Sequence's similarity and genetic distance comparisons with the other sequence data of GenBank and BOLD system supported the accurate identification of the 84 putative species in the present study. The exact or near matches (98%–100%) identity with reference DNA libraries both in BLAST and the BOLD Identification System is a strong indication of the success of the DNA barcoding approach of our study as also found in other studies (Alcantara & Yambot, 2016; Bhattacharjee et al., 2012; Cerutti‐Pereyra et al., 2012; Filonzi et al., 2010; Joly et al., 2014; Kress & Erickson, 2012; Ratnasingham & Hebert, 2007; Ward, 2009; Zhang & Hanner, 2012). The ML tree showed that all identified species formed separate branches without any overlap between species which further indicates that our barcode database is suitable for discriminating reef fishes of SMI in Bangladesh.

The mean genetic distances between individuals within species were 0.34% (COI) and 0.94% (16S rNA), within genera were 12.26% (COI) and 4.72% (16S rRNA), and within families 19.03% (COI) and 12.43% (16S rRNA). Such gradually increased values from species to families are consistent with the patterns of other DNA barcoding studies of marine fishes, such as the K2P values within species, genera, and families were calculated at 0.39%, 9.93%, and 15.46%, respectively for Australian marine fishes (Ward et al., 2005); 0.30%, 6.60%, and 9.91% for Indian marine fishes (Lakra et al., 2011); 0.32%, 17.26%, and 20.10% for the marine fishes of South China Sea (Wang et al., 2012); 0.21%, 5.28%, and 21.30% for the fish species in Rongchey Bay, China (Wang et al., 2018), and 0.34%, 12.14%, and 17.39% for coastal ray‐finned fishes in Vietnam (Thu et al., 2019). These genetic distances within species of less than 2% are in agreement with the species delimitation threshold as proposed by Ward (2009) which further supports the branch of each identified species in this study.

The transition frequencies are relatively more than the transversion frequencies in mitochondrial genes as similarly found in the present study (392 vs. 167 for the COI gene and 303 vs. 164 for 16S rRNA barcode region) and also in other studies by Gojobori et al. (1982), Curtis and Clegg (1984), Wakeley (1994, 1996). It was known that a larger number of transversion pairs than transitions are related to deep divergence and often with sequence saturation (Yang & Yoder, 1999). The mean intraspecific K2P distance of 0.34% for the COI barcode gene region of reef fishes of SMI is higher than that of fish studies from other geographic areas such as 0.10% in South Africa (Cawthorn et al., 2011), 0.312% in Brazil (Ribeiro et al., 2012), 0.32% in turkey (Keskİn & Atar, 2013), 0.28% in Pakistan (Karim et al., 2016), and 0.21% in Taiwan Strait (Bingpeng et al., 2018). On the other hand, opposite findings, that is, the higher K2P distance was also found in some studies such as 0.57% in the fishes of Java and Bali (Dahruddin et al., 2017), and 0.37% in Pampa Plain, Argentina (Rosso et al., 2012).

The fish species collected from SMI and barcoded in this study were found in different categories of global conservation status according to the IUCN Red List of Threatened Species (IUCN, 2020). Among 84 identified species, sixty‐six species (79%) were categorized as Least Concern (LC), three species (4%) were Data Deficient (DD), thirteen species (15%) were categorized as Not Evaluated (NE) while two species (2%) were considered to be under Vulnerable (VU) category. The majority (LC) do not seem to require any additional protection as required for Critically Endangered, Endangered, Vulnerable, or Near Threatened categories (IUCN, 2020). However, ignoring the management of the LC category is also “unsafe” as they make up the majority portion (79%) of the fish. Though the species listed in NE and DD categories have no or limited biological, ecological, or distributional information, it would be sensible to confer this group careful attention, at least until their status is evaluated.

The SMI presents a variety of physiographic features such as rocky platforms, sandy beaches, sand dunes, lagoons, marshes, tombolo, crenulated shorelines, and coral clusters (Hoque et al., 1979; Hossain et al., 2007). Several anthropogenic threats were seen during the present survey such as internecine human intervention in coral reef destruction via indiscriminate anchoring of boats, fishing on coral reef habitats, throwing garbage into the water, and so on. Government and policymakers should come forward to save the marine biodiversity including reef‐associated fishes of this natural treasure of Bangladesh using essential recommendations from different stakeholders. It is also needed immediately to formulate a sustainable strategic plan to manage this lonely coral island to protect its internal biodiversity and the livelihood of the local people. DNA barcode inventory obtained from this study will contribute to making effective monitoring, conservation, and management strategies of fisheries resources of this only coral island of Bangladesh as done in different regions of the world (Ardura et al., 2010; Lewis et al., 2016; Thomsen et al., 2012; Valdez‐Moreno et al., 2012; Weigt et al., 2012).

## AUTHOR CONTRIBUTIONS

**Kazi Ahsan Habib:** Conceptualization (lead); data curation (lead); formal analysis (equal); funding acquisition (lead); investigation (lead); methodology (lead); project administration (lead); resources (lead); software (equal); supervision (lead); validation (lead); visualization (equal); writing – original draft (lead); writing – review and editing (lead). **Md. Jayedul Islam:** Data curation (equal); formal analysis (equal); investigation (equal); methodology (equal); project administration (supporting); resources (equal); software (equal); validation (equal); visualization (equal); writing – original draft (equal); writing – review and editing (equal). **Md. Nazmus Sakib:** Data curation (supporting); formal analysis (supporting); resources (supporting); visualization (supporting). **Parsha Shanjana Brishti:** Data curation (supporting); formal analysis (supporting); resources (supporting); writing – original draft (supporting). **Amit Kumer Neogi:** Data curation (equal); formal analysis (equal); investigation (equal); methodology (equal); resources (equal); software (equal); visualization (equal); writing – original draft (equal); writing – review and editing (equal).

## FUNDING INFORMATION

This research has been carried out by a competitive research grant (CRG) of the National Technology Program Phase II (NATP‐2) project jointly funded by USAID Trust Fund and Bangladesh Government as coordinated by the program implementation unit (PIU) of Bangladesh Agricultural Research Council (BARC).

## CONFLICT OF INTEREST STATEMENT

The author(s) declare that they have no conflict of interest.

### OPEN RESEARCH BADGES

This article has earned Open Materials and Preregistered Research Design badges. Materials and the preregistered design and analysis plan are available at [[insert provided URL(s) on the Open Research Disclosure Form]].

## Data Availability

DNA sequences: Sequence files can be found in the following Github database (Reef fish sequences of SMI, Bangladesh). All sequences and taxonomic files can also be retrieved from the BOLD system (project code: SAU) and NCBI GenBank (accession number given in Table 1) which are accessible to all researchers. All the taxonomic descriptions with their respective Voucher ID are kept at the Aquatic Bioresource Research Laboratory (ABR Lab), Department of Fisheries Biology and Genetics, Sher‐e‐Bangla Agricultural University, Dhaka, Bangladesh, and have public access with permission.
